# Hepatocellular carcinoma: a systems biology perspective

**DOI:** 10.3389/fphys.2013.00028

**Published:** 2013-02-25

**Authors:** Lorenza A. D'Alessandro, René Meyer, Ursula Klingmüller

**Affiliations:** Division Systems Biology of Signal Transduction, DKFZ-ZMBH Alliance, German Cancer Research Centre (DKFZ)Heidelberg, Germany

**Keywords:** HCC, mathematical modeling, network analysis, gene expression profile, proteomic, hepatocytes, liver

## Abstract

Hepatocellular carcinomas (HCCs) have different etiology and heterogenic genomic alterations lead to high complexity. The molecular features of HCC have largely been studied by gene expression and proteome profiling focusing on the correlations between the expression of specific markers and clinical data. Integration of the increasing amounts of data in databases has facilitated the link of genomic and proteomic profiles of HCC to disease state and clinical outcome. Despite the current knowledge, specific molecular markers remain to be identified and new strategies are required to establish novel-targeted therapies. In the last years, mathematical models reconstructing gene and protein networks based on experimental data of HCC have been developed providing powerful tools to predict candidate interactions and potential targets for therapy. Furthermore, the combination of dynamic and logical mathematical models with quantitative data allows detailed mechanistic insights into system properties. To address effects at the organ level, mathematical models reconstructing the three-dimensional organization of liver lobules were developed. In the future, integration of different modeling approaches capturing the effects at the cellular up to the organ level is required to address the complex properties of HCC and to enable the discovery of new targets for HCC prevention or treatment.

## Introduction

**Figure 1:**
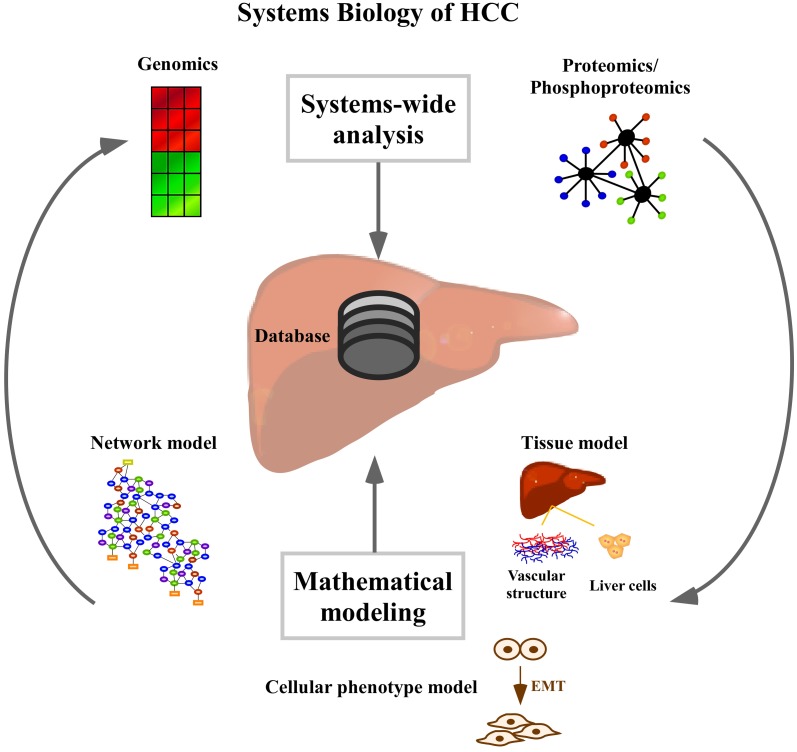
**Systems biology approaches for the study of hepatocellular carcinoma.** Systems biology of HCC at systems-wide level includes the gene expression and proteome profiling and the generation of databases for data storage. Mathematical modeling can describe signaling pathways, the entire network or the organ function by liver tissue models, which include information arising from different levels such as vasculature and cellular level.

Hepatocellular carcinoma (HCC) represents one of the most frequent cancers with the highest incidence in developing countries. Due to its aggressiveness it is third in causing cancer-related deaths worldwide (Ferlay et al., 2010). Major reasons for HCC development are Hepatitis B-virus (HBV) and Hepatitis C-virus (HCV) infection, alcoholic liver diseases, and non-alcoholic fatty liver diseases. Type II diabetes and obesity are among the less common but emerging causes of HCC in Western countries (El-Serag, 2011). Fibrosis and cirrhosis in response to chronic inflammation are characterized by expansion of dysplastic clonal hepatocyte populations (Pons et al., 2005). Dysplasia is characterized by three states: foci of small dysplastic cells, low grade dysplastic nodules characterized by fibrotic tissue, and high grade dysplastic nodules representing preneoplastic lesions. The size of the nodules and their invasiveness define the HCC stage as “early,” “intermediate” or “late.” Primary HCC satellite nodules are characterized by different genomic profiles, and thus contribute to the high heterogeneity of HCC (Cetta et al., 2001). The best treat-ment for HCC is liver transplantation or partial hepatectomy. While the former involves a complete resection of the tumor tissue, its application is limited by the low number of donors and is not advised when metastasis are present. The latter is applied in cases where there is no tumor spread and no cirrhosis, but has limited effects due to only partial resection of the tissue (Befeler and Di Bisceglie, 2002). When none of the previously described methods can be applied, chemotherapy represents a possible choice, although it showed very low efficiency in HCC patients due to genomic heterogeneity (El-Serag, 2011; Lee et al., 2011a) and multidrug resistance of the tumors (Schwartz et al., 2002). Systems biology combines experimental data with mathematical modeling and provides several strategies to deal with the complexity to predict tumor response to targeted therapies. In this review, as schematically shown in Figure 1, we will focus on systems-wide analysis based on high-throughput gene expression and proteomic profiling (Woo et al., 2009; Kim et al., 2011; Lee et al., 2011b) and on different mathematical modeling approaches that can contribute to a deeper understanding of liver functions.

## Systems-wide analysis

High-throughput gene and protein expression profiling provided molecular evidence for the high complexity of HCC. This knowledge allowed screening different stages of HCC, to define subclasses of HCC and to establish criteria for targeted therapies of HCC.

### Genomics

Genomic analysis of HCC samples have been widely performed (Buendia, 2000; Feo et al., 2000) showing that several pathways are altered (Hsu et al., 1991; Zhang et al., 1994; De La Coste et al., 1998) and that the frequency of mutations is variable, thus contributing to the HCC heterogeneity. Several datasets were used to demonstrate a correlation between CGH status, etiology, and histology of HCC (Moinzadeh et al., 2005).

The extensive use of high-throughput gene expression profiling opened the possibility to compare a large number of samples analyzed by different laboratories, enlarging the screening spectrum. Studies have focused on correlations between gene expression profiles and the mutational status of the samples (e.g., p53 status), vascular invasion (Chen et al., 2002; Okada et al., 2003), tumor etiology (e.g., infection status) (Okabe et al., 2001), or early intrahepatic recurrence of the tumor (Iizuka et al., 2003; Kurokawa et al., 2004). High-throughput data require the development of algorithms allowing the identification of genes as predictors. Correlation of gene copy number alteration with gene expression profiles was analyzed for 15 HCC samples by applying a regional pattern recognition approach and Connectivity Map (Woo et al., 2009). The Connectivity Map allows connecting the gene expression profile to drug response (Lamb et al., 2006). A set of 50 genes residing in the most commonly altered regions showed a strong prognostic value, suggesting their potential role as driver genes. The highest prognostic effect has been attributed to 30 genes residing in the 1q and 8q chromosomal arms, known to be involved in the early stages of HCC. Analyses of these effectors of the driver genes revealed a cross-talk among the mTOR, EGFR, and AMPK pathway, and suggested these pathways as candidates for multi-targeted therapy. In a subsequent study, the same authors performed a wider analysis on several gene expression profiles in combination with clinical data (Kim et al., 2011). By combining two independent gene expression signatures associated with HCC recurrence and other clinical parameters, the risk score was calculated based on the Cox coefficient and the expression level. The two datasets showed an overlap of 65 genes representing the risk score factors for predicting overall survival of HCC patients. Interestingly, the enriched functional category of the 65 selected genes was signal transduction. Further experimental validation revealed that Akt, IGFR, and RPS6 phosphorylation as well as a mutation in beta-catenin are strongly associated with the risk score.

A functional genomic study was performed applying siRNA to identify tumor suppressor genes in a mosaic mouse model (p53^−/−^) (Zender et al., 2008). With this approach 12 genes were identified as tumor suppressor genes, such as XOP4, FGF6, and GLO1. Furthermore, miRNA profiling was employed comparing miRNA expression of HCC samples and normal tissue revealing an overall down-regulation of miRNAs (Murakami et al., 2006). Interestingly, the comparison of different HCC stages showed an inverse correlation between the HCC stage and the expression of four miRNAs (*miR*-18, *miR*-20, *miR*-92, and *miR*-99a).

### Proteomics

Gene expression profiles have been employed to derive protein–protein interaction networks of HCC. An improved method that accounts for the topological characteristics of human protein interaction networks (Zhang et al., 2011) was developed to establish a scale-free network based on gene expression data and direct connections between candidate genes and was used to link HCC classifiers to network topology. Integration of the topological feature of the network allowed the selection of candidate genes that strongly affect the tumor classifier performance. The analysis identified 13 HCC-related genes, including members of key signaling cascades, e.g., EGFR, SMAD2, SOCS3, MAPK1, and FOS.

Proteome-wide analysis of the interaction of liver proteins was performed by a yeast-two hybrid technology using 5026 human liver proteins (Wang et al., 2011). This allowed the generation of the human liver protein interaction network (HLPN). To reduce the complexity of the proteome profiling and to discriminate key molecules in HCC, Lee and colleagues (Lee et al., 2011b) employed subcellular fractionation in combination with mass spectrometry analysis comparing normal vs. tumor tissue and identifying 21 potential candidate proteins, such as MATR3 and FASN. To identify disease biomarkers based on phosphorylation differences, quantitative analysis of phosphopeptides from HCC specimen and cell lines were performed (Lee et al., 2009).

Quantitative analysis of the proteome was applied for 11 HCC samples and their healthy counterparts by applying culture-derived isotope tag methods (Li et al., 2012). This approach allows the simultaneous identification and quantification of a large number of proteins. These studies yielded a set of proteins differentially expressed in normal vs. tumor tissue samples and between early and late HCC stages. These results suggested that the granzyme A-mediated apoptosis pathway is upregulated in the HCC samples and could represent a therapeutic target. In summary, the analysis of proteomic profiles is an important prerequisite for an earlier diagnosis and better tumor stage discrimination.

## Databases

The increasing amount of high-throughput data collected in the past years made it necessary to develop appropriate databases in order to facilitate easy queries of genomic alterations or changes in expression levels and to ideally provide a link to clinical patient information. Moreover, databases are essential tools for sharing the available data within the scientific community.

The Cancer Genome Atlas (TCGA) is a database for genomic changes of several cancer types, including 99 HCC samples and their normal liver tissues. TCGA includes gene and miRNA expression data and the epigenetic DNA methylation status.

The integrated Clinical Omics Database (iCOD) collects all available information of 140 cases of HCC, ranging from gene expression profiles to relevant clinical data (Shimokawa et al., 2010). The data can be visualized as a map allowing the user to connect each patient to the molecular, pathological, and clinical features and to correlate gene expression profile with clinical information.

Another source of information is provided by the Liverbase database collecting the results obtained within the Human Liver Proteome Project (HLPP) (Sun et al., 2010). The Liverbase includes information on the human liver at the genomic and proteomic level as well as protein localization and metabolic network data.

The available data of molecular interaction of human chronic liver diseases has been manually curated and collected in the Library of Molecular Association (LOMA) that contains links of gene expression to specific liver diseases (Buchkremer et al., 2010). A potential limiting factor of databases could be a lack of manual curation and of comprehensive annotation. The currently available databases widely overcome these difficulties and represent powerful tools to link intracellular alterations with clinical data.

## HBV- and HCV-induced HCC: a systems biology approach

Although HBV and HCV infection represent one of the major risk factors for HCC development, the molecular mechanisms triggering HBV- or HCV-induced HCC remain to be elucidated.

To understand the central characteristics of each stage of HCC formation with respect to HCV infection, gene expression profiles of 75 HCC samples representing eight different stages of the tumor were compared to normal liver tissue (Wurmbach et al., 2007). Gene signatures for each tumor stage have been identified representing molecular markers for diagnosis. These sets of genes were combined with a HCV specific protein–protein interaction data set that allowed the generation of protein–protein interaction networks of HCV-HCC for each tumor stage (Zheng et al., 2011). The resulting independent networks revealed a higher number of common proteins between the early stages than between the early and later stages of the tumor, indicating a strong deregulation of the network between the precancerous and late tumor stage. This analysis suggested that the adaptive immune response is strongly activated during the cirrhosis phase and down-regulated at the dysplastic nodule stage, while cell cycle progression is constantly up-regulated. Additionally, two non-structural HCV proteins showed to be potential regulators of key components of the networks, suggesting a specific mechanism of action of HCV-HCC.

It was shown that HBV and HCV infection leads to distinct deregulated signaling and gene expression patterns (Iizuka et al., 2002; Honda et al., 2006) that underlie differential features of HCC progression. Comparison of gene expression profiles between HBV or HCV-infected tumor samples were performed and weighted gene co-expression network analysis was applied. Both studies suggested potential targets for therapy to prevent HCC development in cases of viral infection.

## Network models

Network models based on high-throughput data provide an extensive description of HCC but lack information on dynamic properties of the system. Traditionally, kinetic models based on flux balance analysis of enzymatic activities in hepatocytes were developed to describe liver metabolism (Garfinkel and Hess, 1964; Jerby et al., 2010). However, many of the required reaction parameters up to now could not be determined *in vivo*. An alternative approach aiming to generate a human metabolic model was developed based on all available published data (Duarte et al., 2007). This model represents a reference model and has predictive power for metabolite changes, but lacks tissue specificity. A tissue-specific metabolic network model was constructed by applying a constraint-based method and using gene as well as protein expression data from 10 tissues including liver (Shlomi et al., 2008). To reconstruct the human liver metabolism in more detail, Gille and collaborators generated the HepatoNet1 that represents a network based on manually curated biochemical data available in the literature for human hepatocytes (Gille et al., 2010). The HepatoNet1 represents a highly valuable source for metabolic studies of liver function. An even more elaborated model of human liver metabolism was developed by combining gene expression profiles, proteomics, metabolomics, and phenotype data extracted from the literature (Jerby et al., 2010). The described models are based on physiological liver conditions and possess predictive power to simulate metabolic alterations in HCC. Besides the key role of liver metabolism, genomic mutations point to an important role of alterations in signaling networks for the development of HCC. Data-driven discrete logical modeling was applied to compare signaling networks in primary human hepatocytes and four human liver cancer cell lines (Saez-Rodriguez et al., 2011). By stimulating cells with insulin, TNFα, IL6, IL1β, and TGFα, the authors analyzed the activation by phosphorylation of the immediate-early signaling pathways deregulated in HCC. Additionally, to reveal molecular targets for therapeutic applications, combinations of selected kinase inhibitors were applied. By this approach, an interaction regulating Hsp27 phosphorylation specifically present in primary hepatocytes but absent in the screened HCC cell lines has been discovered, known to be associated with tumor progression (Yasuda et al., 2005). In addition, in accordance with previous findings (Hoffmann et al., 2002; Lin and Karin, 2003; Basak et al., 2012), it was shown that NFκB activation in HCC cell lines only requires TNFα stimulation, and that activation of PI3K signaling in response to insulin is predominantly observed in HCC cell lines. In subsequent work, the intracellular response and the secreted molecules induced by inflammatory factors in combination with kinase inhibitors were addressed (Alexopoulos et al., 2010). By applying inference interaction graph methods for network reconstruction, the authors showed that primary hepatocytes were more responsive to inflammatory stimuli, while the HCC cell lines primarily responded to growth factors. The down-regulation of the inflammatory response of HCC cells could be associated with the adaptation of cancer cells to counteract immune surveillance. This work showed that the dissection of network regulation with mathematical modeling requires large numbers of measurements under different conditions.

## Cellular phenotype and liver tissue models

HCC is extremely heterogeneous and therefore the analysis of cellular phenotype is important besides genomic and proteomic studies. The analysis of patient material showed a phenotype suggesting epithelial-mesenchymal transition (EMT) (van Zijl et al., 2011). By treating cells with targeted or cytostatic drugs, the study showed that mesenchymal HCC cells were more resistant to the targeted agents and the combination of both treatments was most potent against both cell types. Based on these observations, a mathematical model employing integro-differential equations was developed (Delitala and Lorenzi, 2012). The integro-differential equations take into account the integral and the derivatives of a function resembling the dynamics of proliferation of epithelial and mesenchymal HCC cells as well as major influencing factors such as mutations, interactions of tumor cells, role of cytokines, and action of cytotoxic and therapeutic agents. By performing model simulations, the authors showed that the highly proliferative cells are selected during cancer progression and that tumor heterogeneity is a cause of resistance to targeted therapy, while cytotoxic drugs are more effective. Therefore, an improved therapeutic strategy has to be developed.

The previously presented studies focused on the cellular level, but an analysis at the organ level is equally important for a comprehensive understanding of the system. By employing image processing, a three-dimensional mathematical center-based (single-cell-based) model of liver lobule was developed (Hoehme et al., 2010). This model includes architectural parameters and process parameters and is able to simulate liver regeneration in response to liver injury in an individual lobule. The model simulations suggested that the liver lobule architecture is primarily restored by proliferation of hepatocytes along the sinusoidal vessels, which are less sensitive to the toxic agent. The model predictions were validated by experimental data based on imaging, confirming the predictive power of the model.

Taken together, these studies show that even for complex situation such as HCC, systems properties can be addressed by combining experimental data with mathematical modeling.

## Conclusions

HCC represents a particularly complex disease and the integration of all features of HCC including the tumor stage, the etiology, the mutational status, the response to therapy, and tumor recurrence are required to better understand its development and to design a most efficient treatment. There is an urgent need for molecular markers specific for HCC to facilitate early diagnosis in order to improve the prognosis after treatment. Systems-wide studies begin to show evidence for HCC classifiers and for the impact of alterations in hub genes for these classifiers. Additionally, first steps have been taken to provide a deeper understanding of dynamic properties of signaling networks in the liver. A summary of the reviewed results is given in Table 1.

**Table 1:** **Summary of the reviewed data and model types**.

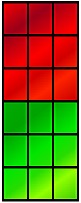	**GENOMICS**	–HCC screening and correlation with clinical data: predictor genes as **risk score factor** –Enriched functional category: **signal transduction** **–siRNA screening:** novel tumor suppressor genes –Correlation **miRNA expression profile** and tumor stage
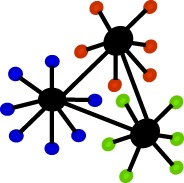	**PROTEOMICS/PHOSPHOPROTEOMIC**	–Link proteomic with genomic: **HCC-related genes/proteins** –Combination of **proteome profiling** and **subcellular protein localization** –Quantitative proteomic: discrimination between **early and late HCC stages** –**HCC biomarkers** based on phosphopeptide analysis –**HCV and HBV-induced HCC**: adaptive immune response and tumor progression
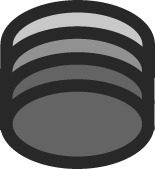	**DATABASE**	**–TCGA:** The Cancer Genome Atlas **–iCOD:** integrated Clinical Omics Database **–Liverbase:** data from the Human Liver Proteome Project **–LOMA:** Library of Molecular Association
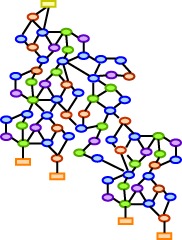	**NETWORK MODEL**	**–Metabolic network** model of primary human hepatocytes –Comparison of **signaling pathway** activation of HCC cell lines with primary hepatocytes –Analysis of intracellular response and molecules secretion upon **inflammatory stimuli**
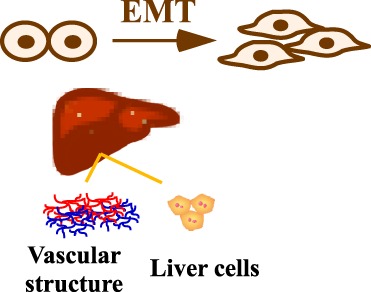	**CELLULAR PHENOTYPE and TISSUE MODEL**	–Mathematical model resembling the **epithelial-mesenchymal transition** phenotype of HCC –Most efficient **therapies:** combination of **targeted and cytotoxic drugs** –Mathematical model based on experimental data resembling single **liver lobule regeneration**

In the table the keywords are highlighted in bold.

Future developments require the integration of data at different scales, connecting the genomic information to the signaling regulation and finally to tumor behavior. To this aim, model integration linking intracellular events to responses at the organ level is essential. The major challenge is to develop mathematical formalisms allowing connecting events occurring at different time scales. In conclusion, the combination of clinical and experimental data with mathematical modeling promises to provide means to handle the complexity that is characteristic for HCC and to facilitate the development of personalized targeted therapy.

### Conflict of interest statement

The authors declare that the research was conducted in the absence of any commercial or financial relationships that could be construed as a potential conflict of interest.

## Research Topic:

From structural to molecular systems biology: experimental and computational approaches to unravel mechanisms of kinase activity regulation in cancer and neurodegeneration

## Topic Editor:

Matteo Barberis, Humboldt University Berlin, Germany; Max Planck Institute for Molecular Genetics, Berlin, Germany
